# An association study of cyclase‐associated protein 2 and frailty

**DOI:** 10.1111/acel.13918

**Published:** 2023-08-03

**Authors:** Silvia Pelucchi, Chiara Macchi, Laura D'Andrea, Paolo Dionigi Rossi, Michela Carola Speciani, Ramona Stringhi, Massimiliano Ruscica, Beatrice Arosio, Monica Di Luca, Matteo Cesari, Valeria Edefonti, Elena Marcello

**Affiliations:** ^1^ Department of Pharmacological and Biomolecular Sciences "Rodolfo Paoletti" Università degli Studi di Milano Milan Italy; ^2^ Geriatric Unit Fondazione IRCCS Ca' Granda Ospedale Maggiore Policlinico Milan Italy; ^3^ General Medicine Hospital San Leopoldo Mandic Merate Italy; ^4^ Department of Clinical Sciences and Community Health, Branch of Medical Statistics, Biometry and Epidemiology "G.A. Maccacaro" Università degli Studi di Milano Milan Italy; ^5^ Department of Cardio‐Thoracic‐Vascular Diseases Fondazione IRCCS Cà Granda Ospedale Maggiore Policlinico Milan Italy; ^6^ Department of Clinical Sciences and Community Health Università degli Studi di Milano Milan Italy; ^7^ Fondazione IRCCS Ca' Granda Ospedale Maggiore Policlinico Milan Italy

**Keywords:** aging, biomarker, cyclase‐associated protein 2, frailty index

## Abstract

Frailty is a geriatric syndrome that results from multisystem impairment caused by age‐associated accumulation of deficits. The frailty index is used to define the level of frailty. Several studies have searched for molecular biomarkers associated with frailty, to meet the needs for personalized care. Cyclase‐associated protein 2 (CAP2) is a multifunctional actin‐binding protein involved in various physiological and pathological processes, that might reflect frailty's intrinsic complexity. This study aimed to investigate the association between frailty index and circulating CAP2 concentration in 467 community‐dwelling older adults (median age: 79; range: 65–92 years) from Milan, Italy. The selected robust regression model showed that circulating CAP2 concentration was not associated with chronological age, as well as sex and education. However, circulating CAP2 concentration was significantly and inversely associated with the frailty index: a 0.1‐unit increase in frailty index leads to ~0.5‐point mean decrease in CAP2 concentration. Furthermore, mean CAP2 concentration was significantly lower in frail participants (i.e., frailty index ≥0.25) than in non‐frail participants. This study shows the association between serum CAP2 concentration and frailty status for the first time, highlighting the potential of CAP2 as a biomarker for age‐associated accumulation of deficits.

## Graphical abstract

Circulating CAP2 concentration is not associated with chronological age, sex and education. CAP2 concentration is significantly and inversely associated with the Frailty Index (a 0.1‐unit increase in Frailty Index leads to ~0.5‐point mean decrease in CAP2 concentration). Mean CAP2 concentration was significantly lower in frail participants than in non‐frail subjects. CAP2 provides a snapshot of the frailty status representing a potential biomarker for age‐related accumulation of deficits.

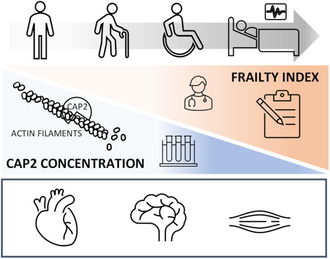

Frailty is a clinically recognized condition characterized by increased vulnerability to stressors due to reduced homeostatic reserve (Clegg et al., 2013). From a biological perspective, frailty is driven by the lifelong accumulation of molecular and cellular deficits that involves different organs (e.g., skeletal muscle and brain) and systems (e.g., respiratory, cardiovascular, and endocrine). Frailty is indicated as the best approach to capture individuals' physiological decline and biological aging (Mitnitski et al., 2001). While there is a general agreement on frailty's theoretical definition, its clinical identification is challenging due to pathophysiological complexities and clinical manifestations (Cesari, Calvani, & Marzetti, 2017). Moreover, multiple frailty definitions exist (Cesari, Marzetti, et al., 2017), and many operational approaches have been proposed over time (Morley et al., 2013). Frailty is most frequently assessed either using the phenotypic model or measuring the accumulation of health deficits through the frailty index (FI). While the frailty phenotype is focused on the physical domain of frailty (Cesari et al., 2014), the FI mirrors the individual's biological age and is expressed by the ratio of an individual's health deficits over the total possible deficits, evaluated within a comprehensive geriatric assessment (Mitnitski et al., 2001).

Several studies searched for frailty biomarkers to improve the understanding of its biological background and potentially support the development of specific interventions. The most studied circulating biomarkers are those related to the inflammatory response (Cannizzo et al., 2011). However, frailty's complex underlying pathophysiology makes identifying specific biomarkers extremely challenging. Most of the proposed frailty biomarkers have been investigated using the phenotypic model, approaching the condition as a physical syndrome. Exploring frailty's biological background, as measured by FI, might provide more information on the multidimensional aging phenomenon by including symptoms, signs, functional impairments, diseases, and laboratory abnormalities.

In this context, we focused on the cyclase‐associated protein 2 (CAP2), an actin‐binding protein that controls actin cytoskeleton dynamics and, thereby, regulates cellular processes such as muscle contraction and changes in synaptic morphology that underlie memory and learning (Rust et al., 2020). Remarkably, CAP2 expression is restricted to a limited number of tissues, including heart, skeletal muscle, and brain (Bertling et al., 2004), which are the organs mostly affected by frailty. CAP2 has been identified as a crucial regulator of the physiological functions of these organs and alterations in CAP2 have been associated to many age‐related diseases, such as Alzheimer's disease (AD) (Rust & Marcello, 2022). For instance, alterations in CAP2 pathway can contribute to synaptic dysfunction and cognitive deficits in AD patients (Pelucchi et al., 2020) and, thereby, CAP2 levels might be related to the neurocognitive component of frailty. Moreover, the relevance of CAP2 in controlling heart and skeletal muscle function has been observed in CAP2 knockout mouse (Field et al., 2015; Kepser et al., 2019) and confirmed by several human genetic studies (Aspit et al., 2019; Gurunathan et al., 2022), suggesting that CAP2 can be associated to sarcopenia, the biological substrate of physical frailty.

Given CAP2 biological functions, we measured its circulating concentration to assess its potential association with frailty, defined by the FI, in Italian older adults.

Participants' median age was 79 years (IQR: 75–83; maximum value = 92). Among participants, 55.7% were women, and 51.8% reported nine or more years of education. Their median FI was 0.22 (IQR: 0.15–0.29; maximum value = 0.57). Finally, their median CAP2 concentration was 5.21 ng/mL (IQR: 3.32–8.26; maximum value = 57.8) (Table S1). No significant differences were found in the percentages of participants with or without the single health deficits included in the FI calculation, according to CAP2 concentration being below or equal to/above its median value (Table S2).

Table 1 provides results on the linear relationship between age, sex, education, and FI (independent variables) and CAP2 concentration (dependent variable) obtained from the selected robust regression models. In the confounding‐factor‐only model (left columns), no socio‐demographic factor was detected to significantly influence CAP2 concentration (all *p* from *t* tests >0.05). When the FI was introduced into the model in continuum (middle columns), it was the only variable significantly and inversely affecting CAP2 concentration (*β* = −0.47 for a 0.1‐unit increment; SE = 0.17; *p* = 0.006). Similarly, when the FI was introduced as a dichotomous variable (non‐frail and frail subjects; right columns), the *β* coefficient was still negative and significantly different from 0 (*β* = −0.73 for frail participants; *p* = 0.03). Based on this model, the frail participants showed a lower mean CAP2 concentration (estimated marginal mean [EMM]: 4.96, 95% confidence interval [CI]: 4.44–5.49 ng/mL) than the non‐frail participants (EMM: 5.69, 95% CI: 5.26–6.12 ng/mL) (Figure 1).

**TABLE 1:** β coefficients (estimate), standard errors (SE), and *p*‐values (*p*, from *t* test testing the null hypothesis that each parameter was equal to 0), as obtained from the selected robust linear regression models assessing the relationship between confounding factors and frailty index (FI) (independent variables) and cyclase‐associated protein 2 (CAP2) concentration (dependent variable).

	Confounding‐factor model			FI‐based model					
				Continuous FI			Dichotomous FI		
	Estimate	SE	*p*	Estimate	SE	*p*	Estimate	SE	*p*
Intercept	8.81	2.46	0.0004	8.27	2.45	0.0008	7.62	2.53	0.003
Chronological age	−0.05	0.03	0.11	−0.03	0.03	0.38	−0.03	0.03	0.35
Sex	0.24	0.32	0.45	0.16	0.32	0.61	0.23	0.32	0.46
Education (6–8 years)	0.62	0.47	0.19	0.61	0.47	0.20	0.60	0.47	0.20
Education (9–13 years)	0.21	0.43	0.63	0.09	0.43	0.84	0.10	0.44	0.81
Education (14–18 years)	0.53	0.51	0.30	0.32	0.51	0.53	0.38	0.52	0.46
FI									
(0.1‐unit increase)	–	–	–	−0.47	0.17	0.006	–	–	–
(Frail participants)	–	–	–	–	–	–	−0.73	0.34	0.03

**FIGURE 1:**
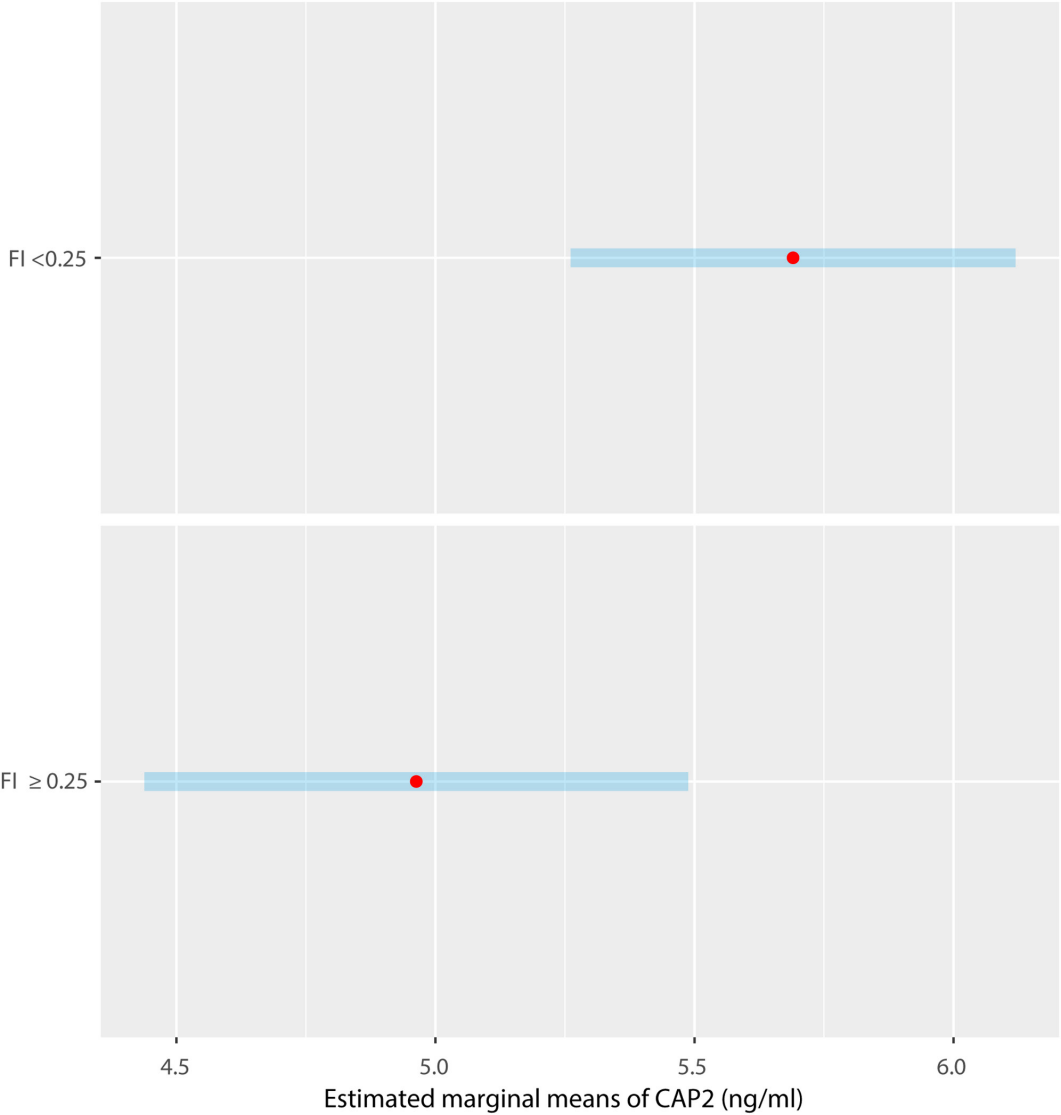
Estimated marginal means of cyclase‐associated protein 2 (CAP2) levels as derived from the robust linear regression model assessing the relationship between confounding factors and frailty index (FI) (dichotomous variable) and CAP2 concentration. Frail subjects were defined as those with a FI ≥0.25.

This study has described for the first time a significant association between FI and circulating CAP2 concentration in a cross‐sectional analysis of older adults, highlighting the potential role of CAP2 as a biomarker of biological aging. While the investigated sociodemographic characteristics (or their interactions) do not significantly modify CAP2, CAP2 circulating concentration was inversely related to FI. In detail, (i) increases in FI in continuum, indicating greater frailty, were related to decreases in serum CAP2 concentration; (ii) mean circulating CAP2 concentration was significantly lower in frail than in non‐frail participants.

Although it is significantly related to FI (after adjusting for chronological age), serum CAP2 concentration is not significantly related to chronological age alone. This suggests a potential role for CAP2 as a biomarker of age‐related accumulation of deficits that cannot be mirrored by chronological age. Indeed, chronological age can be considered inadequate for capturing individuals' homeostatic weakness, increased vulnerability to diseases, and decreased physiological integrity associated with aging.

Our results support the hypothesis that circulating CAP2 concentration provides a snapshot of frailty. Although the FI provides a multidimensional measure of clinical phenotypes related to deficits, biomarkers such as CAP2 may assist in better targeting at‐risk subpopulations, thus (i) ameliorating planning and implementation of personalized preventive and therapeutic interventions and (ii) supporting a more accurate prediction of frailty's trajectory. In addition, several studies have suggested that the age‐associated decline in adaptive homeostasis is a major risk factor for many age‐associated diseases, such as cancer, cardiovascular disease, and AD (Cannizzo et al., 2011). Therefore, CAP2 as a biomarker of frailty could be used to filter participants recruited for clinical trials, maximizing the intervention's effect size by selecting individuals particularly at risk.

The strengths of our analysis are the multidimensional assessment of older adults and the use of robust regression models to account for heteroscedasticity of the error term and outliers. The main limitations of our study are its cross‐sectional nature, the lack of a validation in another sample, and the possible influence of other unmeasured lifestyle factors (Romero‐Ortuno, 2014).

Future studies are needed to (i) validate diagnostic performance of CAP2 in larger samples alone or combined with other biomarkers and (ii) establish the potential prognostic value of CAP2 in frailty and its possible role in tracking the efficacy of clinical interventions in frail older individuals.

## 1 EXPERIMENTAL PROCEDURES

### 1.1 Study design

At the geriatric outpatient clinic, Fondazione IRCCS Ca′ Granda Ospedale Maggiore Policlinico, Milan, Italy, 467 community‐dwelling older adults underwent a comprehensive geriatric assessment and provided biological specimens between 2005 and 2020 (Appendix S1 for additional details). A 46‐item FI was defined (Table S3) based on the geriatric evaluation, to include multimorbidity and disability phenomena, according to standardization criteria (Searle et al., 2008).

### 1.2 CAP2 measurement

Serum CAP2 concentration was determined using a commercially available ELISA kit (n. IK5163; Immunological Sciences).

### 1.3 Statistical analysis

Violations of the standard ordinary least squares assumptions suggested the adoption of the robust MM estimator for our analyses, in which possible confounding factors (chronological age [continuous], sex [categorical], and education [categorical]) and FI (continuous or categorical) were considered as independent variables and regressed on the dependent variable CAP2. Based on likelihood ratio test *p‐values* >0.1 from models including up to the three‐way interaction, the final confounding‐factor‐only model included the main effects for chronological age, sex, and education. Based on likelihood ratio test *p‐values* >0.1 from models including up to the four‐way interaction, the final selected FI‐based model (FI continuous) included terms for chronological age, sex, education, and FI. The same robust model was also fitted using a dichotomized FI variable (categorical: non‐frail and frail subjects) and the EMMs were calculated by averaging predictions from this model over a reference grid (Lenth, 2021). Details are provided in the Appendix S1. Calculations were performed using the open‐source statistical computing environment R (R Development Core Team, 2022) with libraries *MASS* (Venables & Ripley, 2002) and *emmeans* (Lenth, 2021).

## AUTHOR CONTRIBUTIONS

S.P. and C.M. designed, conducted, and analyzed the experiments, revised the manuscript; L.D.A. conducted and analyzed the experiments; P.D.R. revised the manuscript, funding acquisition; M.C.S. analyzed the experiments, revised the manuscript; R.S. analyzed the experiments, revised the manuscript; M.R. designed experiments, revised the manuscript; B.A. designed experiments, revised the manuscript; M.D.L. revised the manuscript; M.C. designed experiments, revised the manuscript; V.E. performed statistical analysis, wrote and revised the manuscript; E.M. conceptualization, funding acquisition, project administration, wrote and revised the manuscript.

All authors read and approved the final manuscript.

## FUNDING INFORMATION

This work was supported by the Fondazione Cariplo (grant number 2018‐0511 to EM and P.D.R), the Italian Ministry of University and Research (grant numbers PRIN 2017MYJ5TH and PRIN 20202THZAW to MDL, PRIN 2017B9NCSX and PRIN 202039WMFP to EM, MUR Progetto Eccellenza).

## CONFLICT OF INTEREST STATEMENT

The authors declare that they have no competing interests.

## ETHICS STATEMENT

All participants provided informed consent to use their clinical and biological data for the research (study protocol number 0001669, January 16, 2020).

## Supporting information


Appendix S1.

## Data Availability

All data generated or analyzed during this study are included in this published article and its supplementary information files. The dataset analyzed during the current study are available from the corresponding authors on reasonable request.
