# Supplementary material for: An association study of cyclase‐associated protein 2 and frailty
Source: Aging Cell. 2023 Aug 3;22(9):e13918. doi: 10.1111/acel.13918 (PMC10497846; doi:10.1111/acel.13918)
Supplement: Supplementary file 1 — Appendix S1. [file ACEL-22-e13918-s001.docx]

**Supplementary Tables**

**Supplementary Table 1**. Main characteristics of the study sample (n=467). Participants’ main characteristics were explored using descriptive statistics, including medians, means, and quartiles for continuous variables and absolute frequencies and percentages for categorical variables.

| **Main Characteristics** | | |
| --- | --- | --- |
| **Chronological age (years)** | Minimum | 65 |
|  | 1st Quartile | 75 |
|  | Median | 79 |
|  | Mean | 78.83 |
|  | 3rd Quartile | 83 |
|  | Maximum | 92 |
| **Sex** | Male | 207 (44.33%) |
|  | Female | 260 (55.67%) |
| **Education (years)** | 0-5 years | 104 (22.27%) |
|  | 6-8 years | 121 (25.91%) |
|  | 9-13 years | 167 (35.76%) |
|  | 14-18 years | 75 (16.06%) |
| **Frailty Index** | Minimum | 0.00 |
|  | 1st Quartile | 0.15 |
|  | Median | 0.22 |
|  | Mean | 0.22 |
|  | 3rd Quartile | 0.29 |
|  | Maximum | 0.57 |
| **CAP2 (ng/ml)** | Minimum | 0.73 |
|  | 1st Quartile | 3.32 |
|  | Median | 5.21 |
|  | Mean | 6.76 |
|  | 3rd Quartile | 8.26 |
|  | Maximum | 57.8 |

**Supplementary Table 2.** Percentages of the study population showing each of the health deficits included in the frailty index (“0”: no deficit or “1”: deficit present) overall and by dichotomous cyclase-associated protein 2 (CAP2), expressed as below or equal to/above its median value. The Chi square test of independence was used to assess if percentages of participants with/without each disease differ according to CAP2 being below or equal to/above its median value; when a single cell showed an absolute frequency smaller than 10 (i.e., congestive heart failure), a two-sided Fisher’s exact test was used instead of the Chi square test.

| **Diseases** | | **Overall sample** | **CAP2** | | **p-value** |
| --- | --- | --- | --- | --- | --- |
|  |  |  | **< median value** | **≥ median value** |  |
| **Hypertension** | **0** | 34.9% | 36.2% | 33.6% |  |
|  | **1** | 65.1% | 63.8% | 66.4% | 0.557 |
| **Diabetes** | **0** | 85.7% | 87.9% | 83.4% |  |
|  | **1** | 14.3% | 12.1% | 16.6% | 0.163 |
| **Congestive heart failure** | **0** | 97.5% | 97.2% | 97.9% |  |
|  | **1** | 2.5% | 2.8% | 2.1% | 0.755 |
| **Coronary heart disease** | **0** | 85.7% | 83.2% | 88.1% |  |
|  | **1** | 14.3% | 16.8% | 11.9% | 0.131 |
| **Cardiac arrhythmia** | **0** | 92.6% | 93.0% | 92.1% |  |
|  | **1** | 7.4% | 7.0% | 7.9% | 0.736 |
| **Chronic obstructive pulmonary disease** | **0** | 89.7% | 90.1% | 89.4% |  |
|  | **1** | 10.3% | 9.9% | 10.6% | 0.797 |
| **Decreased visual acuity** | **0** | 52.5% | 54.9% | 49.7% |  |
|  | **1** | 47.5% | 45.1% | 50.3% | 0.300 |
| **Hearing loss** | **0** | 74.1% | 72.6% | 75.9% |  |
|  | **1** | 25.9% | 27.4% | 24.1% | 0.441 |
| **Osteoarthritis** | **0** | 58.7% | 58.6% | 58.7% |  |
|  | **1** | 41.3% | 41.4% | 41.3% | 0.982 |
| **Vascular endothelial abnormalities** | **0** | 49.0% | 53.0% | 44.5% |  |
|  | **1** | 51.0% | 47.0% | 55.5% | 0.0865 |
| **Chronic renal insufficiency** | **0** | 89.7% | 87.9% | 91.6% |  |
|  | **1** | 10.3% | 12.1% | 8.4% | 0.220 |
| **Hepatopathy** | **0** | 79.1% | 80.9% | 77.0% |  |
|  | **1** | 20.9% | 19.1% | 23.0% | 0.327 |
| **Depression** | **0** | 58.0% | 55.6% | 60.4% |  |
|  | **1** | 42.0% | 44.4% | 39.6% | 0.291 |
| **Cerebrovascular disease** | **0** | 44.3% | 43.3% | 45.5% |  |
|  | **1** | 55.7% | 56.7% | 54.5% | 0.642 |
| **Cognitive impairment** | **0** | 89.1% | 87.5% | 90.6% |  |
|  | **1** | 10.9% | 12.5% | 9.4% | 0.277 |
| **Cancer** | **0** | 80.4% | 80.0% | 80.8% |  |
|  | **1** | 19.6% | 20.0% | 19.2% | 0.829 |
| **Osteoporosis** | **0** | 75.4% | 75.0% | 75.7% |  |
|  | **1** | 24.6% | 25.0% | 24.3% | 0.852 |
| **Anemia** | **0** | 92.4% | 92.6% | 92.1% |  |
|  | **1** | 7.6% | 7.4% | 7.9% | 0.876 |
| **Diverticulosis** | **0** | 82.0% | 82.3% | 81.7% |  |
|  | **1** | 18.0% | 17.7% | 18.3% | 0.865 |

**Supplementary Table 3.** List of the 46 biochemical and health deficits included in the frailty index. Each item of the frailty index was scored as “0” (no deficit) or “1” (deficit present). The frailty index was calculated as the number of deficits presented by the individual divided by the total number of deficits considered in the evaluation. While the frailty index is calculated on a continuous scale, its values can be grouped to summarize and compare different health conditions. Here, participants with a frailty index ≥0.25 are defined as frail.

| **Deficit** |
| --- |
| **Biochemical parameters**  Cholesterol >200 mg/dl  CRP >0.5 mg/dl  Vitamin B12 <191 ng/l; Vitamin B12 >663 ng/l  Folate <4.6 µg/l; Folate >18.7 µg/l  25-OH Vitamin D <30 µg/l  TSH <0.28 mIU/l; TSH >4.30 mIU/l  **Signs**  Pain  Bowel incontinence  Sleep disorders  BMI <21 kg/m^2^; BMI >30 kg/m^2^  Edema  Tremor  **Disabilities**  Mobility impairment  ADL - disability in self-feeding  ADL - disability in dressing  ADL - disability in bathing  ADL - disability in transferring  ADL - disability in toileting  ADL - incontinence  IADL – disability in using telephone  IADL - disability in shopping  IADL - disability in food preparation  IADL - disability in housekeeping  IADL - disability in doing laundry  IADL - disability in travelling by car or public transportation  IADL - disability in medication use  IADL - disability in handling finances  **Diseases**  Hypertension  Diabetes  Congestive heart failure  Coronary heart disease  Cardiac arrhythmia  Chronic obstructive pulmonary disease  Decreased visual acuity  Hearing loss  Osteoarthritis  Vascular endothelial abnormalities  Chronic renal insufficiency  Hepatopathy  Depression  Cerebrovascular disease  Cognitive impairment  Cancer  Osteoporosis  Anemia  Diverticulosis |

CRP: C-reactive Protein, TSH: Thyroid-Stimulating Hormone, BMI: Body Mass Index, ADL: Activity of Daily Living, IADL: Instrumental Activity of Daily Living.

1. **Supplementary Experimental Procedures**

**Study design**

This study was carried out at Fondazione IRCCS Ca’ Granda Ospedale Maggiore Policlinico, Milan, Italy between 2005 and 2020 and included 467 community-dwelling older adults (65-92 years old) who were consecutively admitted at the geriatric outpatient clinic and provided biological specimens stored in the outpatient clinic’s biobank. All participants were admitted to the geriatric unit for the investigation of a suspect cognitive decline, mostly reported by the general practitioner. All subjects referring to the outpatient clinic in the identified timeframe were included in the study according to the following inclusion criteria: (i) age of 60 years or older and (ii) willingness to participate and provide written informed consent. Subjects presenting the following criteria were excluded: (i) life threatening illness or estimated life expectancy lower than 6 months; (ii) cognitive impairment defined as a Mini Mental State Examination (MMSE) <24/30; (iii) clinical judgement about safety or adherence issues.

The current analysis was based on the initial geriatric examination that included a multidimensional geriatric assessment providing information on medical history, and cognitive, functional, and physical status of the recruited subjects. In detail, the cognitive function was assessed by using a modified version of the MMSE (Magni et al. 1996) and the presence of depression was evaluated by using the Geriatric Depression Scale. The functional status was assessed by using the Activity of Daily Living and the Instrumental Activity of Daily Living scales (Graf 2008). The mobility impairment is defined based on anamnestic data.

**Cyclase-associated protein 2 measurement**

Overnight fasting blood samples were obtained from participants in the morning. Serum was obtained after clotting and centrifugation at 1200g for 15 min at 4 °C, rapidly frozen, and stored at -80 °C. The serum samples were diluted at 1:10, and their cyclase-associated protein 2 (CAP2) concentration was determined by using a commercially available enzyme-linked immunosorbent assay kit (catalog number IK5163; Immunological Sciences, Rome, Italy). This assay has high sensitivity and specificity for CAP2 detection; no significant cross-reactivity or interference between CAP2 and its analogs was observed. The intra-assay coefficient of variability was <8%, and the inter-assay coefficient of variability was <10%.

**Statistical analysis**

Violations of the standard ordinary least squares assumptions suggested the adoption of the robust MM estimator for our analysis, in which possible confounding factors and frailty index (FI) (independent variables) were regressed on CAP2 (dependent variable). In the absence of previous literature on possible determinants of CAP2 except for a previous paper of our group (Pelucchi et al. 2020), we chose as confounding factors those variables traditionally related with FI and available in our dataset. In detail, candidate variables from literature inspection were chronological age, sex, and education (e.g., Gordon et al. 2017; Zimmer et al. 2021). We also preliminary checked if these variables were related to FI in our dataset, by using single and multiple regression models where FI is the dependent variable and possible confounding factors the independent ones. After reassuring checks on their effect on FI, we first fitted a confounding-factor-only model, by including chronological age (continuous), sex (categorical), and education (categorical), alone, in pairs, all three together, and after adding all two-way and three-way interactions. Based on likelihood ratio test p-values > 0.1, the final confounding-factor-only model included the main effects for chronological age, sex, and education. Then we considered a FI-based model where FI (continuous) was added to the final confounding-factor-only model. Based on likelihood ratio test p-values > 0.1 for all the available two- and three-way interactions involving FI and the four-way interaction, the final selected FI-based model included terms for chronological age, sex, education, and FI. In addition, we fitted the same robust model using a dichotomized FI variable (categorical: non-frail and frail subjects). We calculated the EMMs by averaging predictions from this model over a reference grid (Lenth, 2021). Calculations were performed using the open-source statistical computing environment R (R Development Core Team, 2022) with libraries MASS (Venables & Ripley, 2002) and emmeans (Lenth, 2021).

References

Gordon EH, Peel NM, Samanta M, Theou O, Howlett SE & Hubbard RE (2017) Sex differences in frailty: A systematic review and meta-analysis. *Exp Gerontol* 89, 30–40.

Graf C (2008) The Lawton instrumental activities of daily living scale. *Am J Nurs* 108, 52–62; quiz 62–3.

Magni E, Binetti G, Bianchetti A, Rozzini R & Trabucchi M (1996) Mini-Mental State Examination: a normative study in Italian elderly population. *Eur J Neurol* 3, 198–202.

Pelucchi S, Vandermeulen L, Pizzamiglio L, Aksan B, Yan J, Konietzny A, Bonomi E, Borroni B, Padovani A, Rust MB, di Marino D, Mikhaylova M, Mauceri D, Antonucci F, Edefonti V, Gardoni F, di Luca M & Marcello E (2020) Cyclase-associated protein 2 dimerization regulates cofilin in synaptic plasticity and Alzheimer’s disease. *Brain Commun* 2(2):fcaa086.

Zimmer Z, Saito Y, Theou O, Haviva C & Rockwood K (2021) Education, wealth, and duration of life expected in various degrees of frailty. *Eur J Ageing* 18, 393–404.
